# Experimental Realization of Extreme Heat Flux Concentration with Easy-to-Make Thermal Metamaterials

**DOI:** 10.1038/srep11552

**Published:** 2015-06-25

**Authors:** Fei Chen, Dang Yuan Lei

**Affiliations:** 1 Department of Applied Physics, The Hong Kong Polytechnic University, Hong Kong, China

## Abstract

The ability to harvest thermal energy and manipulate heat fluxes has recently attracted a great deal of research interest because this is critical to achieve efficient solar-to-thermal energy conversion in the technology of concentrated solar thermal collectors. Thermal metamaterials with engineered thermal conduction are often utilized to control the diffusive heat flow in ways otherwise not possible with naturally occurring materials. In this work, we adopt the transformation thermodynamics approach to design an annular fan-shaped thermal metamaterial which is capable of guiding heat fluxes and concentrating thermal energy to the central region of the metamaterial device without disturbing the temperature profile outside the structure – a fascinating and unique feature impossibly achieved with homogeneous materials. In experiment, this rationally-designed metamaterial structure demonstrates extreme heat flux compression from both line-shaped and point thermal sources with measured concentration efficiency up to 83.1%, providing the first experimental realization of our recent theoretical prediction (T. Han *et al.*, *Energy Environ. Sci.*, 2013, 6, 3537-3541). These unprecedented results may open up new possibilities for engineering thermal materials with desired properties that can be used for dramatically enhancing the efficiency of the existing solar thermal collectors.

The utilization of renewable energy has significantly developed in the past few decades because the fossil fuels, also the most notorious culprits of environmental pollution, are being depleted^1^,^2^. Solar energy has been widely recognized to have unbelievable potential as one of the most important renewable energy sources. As a result, solar cells are playing an increasingly important role in converting solar energy to electricity in industrial production, daily life, and even in aerospace exploration^3^,^4^,^5^,^6^,^7^,^8^. In general, the conversion of solar energy into electricity can be achieved *via* two approaches, namely direct solar-electrical energy conversion with the use of the aforementioned solar cell devices^3^,^4^,^5^,^6^,^7^,^8^ and indirect conversion with thermal energy as a mediator (collected by a device known as a solar thermal collector) that drives a heat engine to generate electricity^9^,^10^,^11^. In addition, solar thermal collectors such as flat plate thermal systems are also widely and simply used for water heating in our daily life. A critical issue in the design of an efficient solar thermal collector is to maximize the collection efficiency and guide the harvested heat energy to desired positions for subsequent use either by a heat engine or for simple water heating^9^,^10^,^11^.

Thermal metamaterials are a class of artificial composite structures with engineered thermal conduction and are often utilized to control the diffusive heat flow in ways otherwise impossible with naturally occurring materials^12^. This concept has recently been proposed by realizing the fact that mathematical treatments used in transformation Maxwell’s equations^13^,^14^,^15^,^16^ can be adapted to the Fourier heat equation^17^,^18^, forming a new research branch named transformation thermodynamics^19^,^20^,^21^. Using the transformation thermodynamics approach, thermal metamaterials have recently demonstrated unprecedented abilities and striking effects such as thermal cloaking^20^,^21^,^22^,^23^, thermal concentration and rotation^20^,^24^,^25^, thermal transparency^26^, to name a few here. Note that, by using the scattering-cancellation method or a scheme derived directly from the conduction equation, thermal cloaking has recently also been demonstrated with isotropic bulk materials^27^,^28^. In the context of efficient concentration of heat fluxes, the pioneering experimental work by Sato *et al.* has shown that their device is capable of concentrating heat current flowing through two annular layers of the compression region^20^. However, the concentration efficiency achieved so far is relatively low with an optimal value of 12% and the materials used in the experiment (agar-water, latex rubber and silicone elastomers containing boron nitride particles) have weak guiding significance for large-scale practical applications in industrial production. Though the thermal invisibility and flux concentration can be realized using microstructured thermal metamaterials^19^, it places many challenging fabrication issues such as harsh inhomogeneity or high anisotropy^21^, an analogous bottleneck inherited from the transformation optics approach adopted in electromagnetic wave dynamics^29^,^30^. Thus, using industrially-feasible natural materials to fabricate thermal concentrators for efficient thermal energy harvesting with less thermal losses is still an important challenge for the development of concentrating solar power technologies.

Very recently, we have theoretically proposed a three-dimensional thermal metamaterial based concentrator with adjustable thermal conduction anisotropy designed by the transformation thermodynamics approach. The thermal-energy harvesting efficiency of the proposed device may achieve 100%^31^, independent of geometrical size and significantly superior to the concentrating devices reported so far. In this paper, we modify the original transformation thermodynamic approach and report on the experimental realization of a simplified two-dimensional analogy that can realize extreme heat flux concentration with record high efficiency but without incurring perturbation to the temperature distribution outside the structure. Within the concentration region, the heat fluxes from both line-shaped and point thermal sources can be efficiently guided and directed to a desired position (the center of the device) without thermal energy losses, demonstrating experimental concentration efficiency up to 83.1% and an increased temperature gradient two times the applied gradient.

## Results

### Transformation thermodynamics concept designing thermal energy concentrator

In the absence of a heat source, the stationary thermal conduction equation is written as ∇·(*κ*∇*T*) = 0, where *κ* is the thermal conductivity and *T* represents the temperature. Upon application of the coordinate transformation originally used in transformation optics studies^13^,^14^, the thermal conduction equation in the transformed space can be rewritten as 
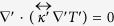
, where in a polar coordinate system.


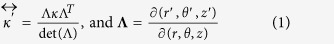


**Figure 1:**
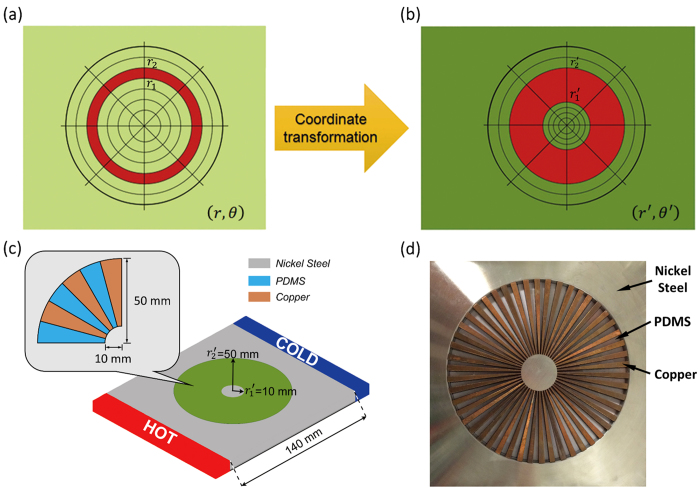
(**a**–**b**) Spatial coordinate transformation design of an efficient thermal concentrator by radially stretching the red annular region *r*_2_ > *r* > *r*_1_ in the virtual space (**a**) to the region 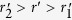 in the physical space (**b**). As *r*_2_ → *r*_1_, the energy concentration or compression efficiency in both regions approaches unity. (**c**) Schematic design of a thermal concentrator where the green area is the compression region (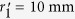 and 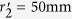) and the gray area is the background with 140-mm side length. (**d**) Photograph of the experimental thermal concentrator device made of alternating layers of copper and PDMS atop a nickel steel plate.

Figure 1 illustrates the transformation thermodynamics concept for designing an efficient thermal concentrator (with an ideal harvesting efficiency up to 100%), which is achieved by radially expanding an infinitely-thin annular region *r*_1_ < *r* < *r*_2_, Fig. 1a) in the virtual space to a finitely-thick annular region (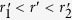, Fig. 1b) in the physical space. As a result of the radial stretching, the coordinates (*r*′, *θ′*, *z*′) in the physical space can be mapped to (*r*, *θ*, *z*) in the virtual space following the equations


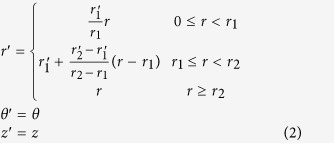


Based on the transformation matrix shown in Eq. (1), the normalized thermal conductivity tensor in the physical space is given as


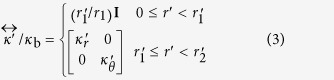


where *κ*_b_ is the uniform thermal conductivity in the virtual space and **I** is a 2 × 2 identity matrix. It can be easily shown that, if we fix 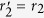 and *r*_2_ − *r*_1_ = *δ* (where *δ* is a small positive value), we will need 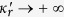 and 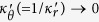 as the value of *δ* is reduced. This high anisotropy in thermal conductivity can be realized by building a stacked composite from macroscopic layers of isotropic materials such as a composite made of alternating sheets of two different materials, one with a thermal conductivity much larger than the other^20^. Because the two alternating conductances add in series in the azimuthal direction whereas they add in parallel in the radial direction, the overall thermal conductivity of the effective medium becomes anisotropic and the heat flux flow changes direction following a particular path of interest^20^.

Figure 1c shows an annular fan-shaped device consisting of alternating layers of A and B with different homogeneous isotropic thermal conductivities *κ*_A_ and *κ*_B_, which can be calculated respectively as 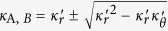 based on Eq. (3). For the layers A and B, copper 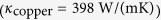 and polydimethylsiloxane (PDMS, *κ*_PDMS_ = 0.27 W/(mK)) are chosen in our experiment because they are more industrially preferable than other materials. Based on the thermal conductivities of copper and PDMS, *κ*_*r*_′ and *κ*_*θ*_′ are calculated to be 199 and 0.540, giving rise to extraordinarily high anisotropy as discussed above. For the device to blend in a host background thermally and not perturb the external thermal profile, the thermal resistance of the host material should be close to the reduced average of those of the two constitute materials in accordance with the effective medium approach, i.e. 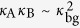, where *κ*_bg_ is the thermal conductivity of the host background material. Based on this requirement, nickel steel (40% Ni) with a thermal conductivity *κ*_bg_ = 10 W/(mK) is chosen as the host background. The following considerations have been taken into account when selecting the conductor and insulator materials for A and B. On the one hand, though silver (*κ*_silver_ = 427 W/(mK)) has an appropriate thermal conductivity, the expensive material cost makes it unsuitable for industry-scale production. The melting point of aluminum (~660 °C) is much lower than the other metals like silver (~961 °C) and copper (~1084 °C) and this renders aluminum not resistant to devices working at high temperatures. On the other hand, both agar-water (*κ*_agar−water_ = 0.56 W/(mK)) and glass (*κ*_glass_ = 0.14 − 0.8 W/(mK)) have relatively larger thermal conductivities than PDMS and latex rubber (*κ*_rubber_ = 0.13 W/(mK)), and PDMS is more industrially preferable than latex rubber. Figure 1d shows a photograph of the fabricated thermal concentrator device consisting of 50 copper wedges and 50 PDMS wedges on a nickel steel plate of 140-mm side length. The inner and outer radii of the wedges are 10 mm and 50 mm as designed in Fig. 1c. Note that Eq. (3) also indicates that the thermal conductivity of the material in the core region 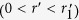 is different from the background material. However, we have observed in both simulation and experiment that the use of the same material for the core as the background still results in very high concentration efficiency. Therefore, for simplicity we choose the same material for the core material (nickel steel) as the background to achieve a balance between the device performance and fabrication complexity.

### Numerical examination of extreme heat concentration without incurring perturbation to external environment

**Figure 2:**
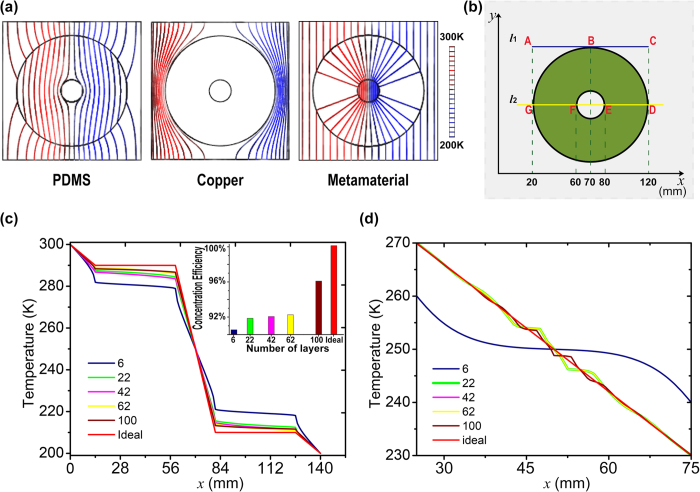
(**a**) Simulated spatial distribution of isothermal lines for the three structures with the annular regions made of PDMS (left), copper (middle) and the metamaterial (right), respectively. The colors of the lines represent the temperature profile. In the simulation, the metamaterial has a set of thermal conductivities 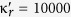 and 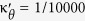. (**b**) In a thermal energy concentrator, we pick the temperatures at seven points (A–G) along the blue and yellow lines both parallel to *x*-axis. The blue line is tangent to the outside circle of the shell at point B and the yellow line crosses the shell and intersects at points D-G. (**c**–**d**) The temperature distribution along the lines GD (c) and AC (**d**) is captured to calculate the harvesting efficiency of thermal energy (inset in (**c**)) and evaluate the degree of perturbation to external temperature profile (**d**) as a function of the total number of the copper and PDMS layers.

To understand the functionality of the designed device, numerical simulation based on the finite-element method and implementation of the stationary thermal conduction equation was performed with commercial software, COMSOL Multiphysics. Figure 2a compares the thermal energy harvesting performance of the three hollow cylindrical devices made of PDMS, copper and the thermal metamaterial, respectively. The former two materials have isotropic thermal conductivities as mentioned above whereas the thermal metamaterial has highly anisotropic thermal conductivities, namely 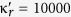 and 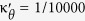. The temperature profile for each device was simulated by maintaining the left and right boundaries at 300 K and 200 K, respectively. It can be seen from the left panel of Fig. 2a that the isothermal lines can easily extend through the PDMS annulus with slight bending and a similar density as the outside region, showing no thermal concentration effect. In sharp contrast, the middle figure shows that the annulus made of copper generates strong bending of the isothermal lines towards the annulus from both hot and cold sides due to the extremely high thermal conductivity of copper. This indicates that the device can attract the heat fluxes and confine them within the annulus, which makes the inner region free of thermal gradient by effective neutralization of high and low temperatures and incurs strong perturbation to the thermal profile outside the annulus. Compared to the structures made of PDMS and copper, the thermal metamaterial device shows two distinctively different features. Firstly, the isothermal lines can penetrate through the metamaterial device within which they are significantly bent towards the inner region 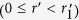, clearly demonstrating strong concentration of the heat fluxes. More quantitatively, the density of the isothermal lines in the inner region is four times that outside the annulus 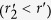. Secondly, the temperature gradients outside the annulus (without any perturbation) and at the inner region (without any neutralization) are uniform, which is an important feature for practical applications and not possible for an annulus made of an isotropic material different from the background.

To quantitatively evaluate the concentration ability of the metamaterial device, we plot the simulated temperature distribution along a horizontal line across the center of the device (see the line DG in Fig. 2b) and use the temperatures recorded at the points D, E, F, and G to calculate the efficiency by the equation 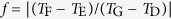. Figure 2c shows the temperature distribution profile recorded as a function of the total number of the copper and PDMS layers and the inset shows the calculated concentration efficiency, with both results compared to that for an ideal metamaterial device. It can be seen that a total number of 100 layers gives rise to a thermal concentration efficiency up to 96.3%, very close to that of 100% for the ideal metamaterial device. The remarkable thermal energy compression behavior can be easily understood by the fact that the temperature gradient at the inner region is significantly larger than that of the other regions or the applied gradient as shown in Fig. 2c. Detailed discussions with respect to temperature gradient will be made in the following session. To further investigate the degree of perturbation to the temperature profile outside the metamaterial concentrator, Fig. 2d shows the temperature distribution along a horizontal line tangent to the boundary of the device (see the line AC in Fig. 2b) as a function of the total number of the copper and PDMS layer. The results demonstrate that a total number of 100 layers produce the least distortion in the temperature profile compared to that recorded for the ideal metamaterial device. These parameters guide our experimental fabrication of the PDMS-copper composite metamaterial concentrator as shown in the following session.

### Experimental realization of efficient heat energy harvesting

In this session we investigate the thermal concentration performance of the metamaterial device as shown in Fig. 1d and compare the experimental results with simulations. The hollow cylinder consists of 100 alternating copper and PDMS wedge-shaped layers with equal dimensions and their inner and outer radii are 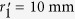 and 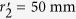, respectively. The whole device is cast into a square-shaped nickel steel of 140-mm side length and covered by a PDMS layer of 100-μm thick in order to capture a high quality image. In experiment, the left boundary of the thermal concentrator device was tightly fixed at a thermostatic heating plate (TSHP) at constant temperature 100 °C and the right boundary was fixed in an ice-water mixture at constant temperature 0 °C, generating two line-shaped thermal sources. The point heat source is simply a corner of the TSHP. The temperature profile of the device was recorded by an infrared camera (NEC^@^TH9100PMV).

**Figure 3:**
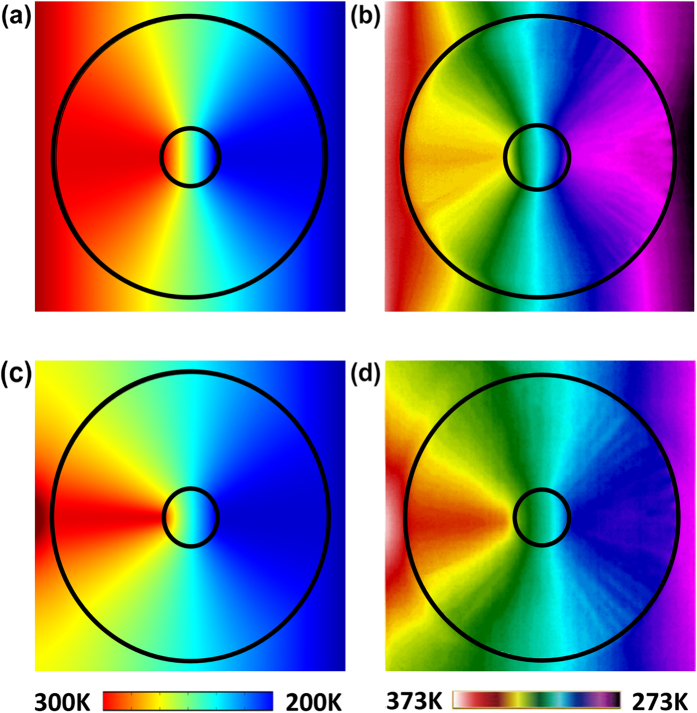
(**a**,**c**) Simulated and measured (**b**,**d**) temperature profile for the fabricated thermal concentrator within a uniform thermal field (**a**,**b**) and in the presence of a point heat source (**c**,**d**). The uniform thermal field is produced by placing two thermostatic line-shaped hot (300 K in (**a**,**c**) and 373 K in (**b**,**d**)) and cold (200 K in (**a**,**c**) and 273 K in (**b**,**d**)) sources nearby the left and right boundaries of the device, respectively. The point heat source is generated by placing a corner of the thermostatic heating plate at the left boundary of the device and emits heat fluxes with cylindrical fronts.

Fig. 3b shows the measured temperature profile in the metamaterial device when the two line-shaped thermal sources are applied. It can be seen that the compression layer attracts the isothermal lines and thereby increases their density inside the annulus and at inner region, which agrees perfectly with the simulated temperature profile as shown in Fig. 3a. More importantly, the temperature gradients outside the annulus and at the inner region keep uniform as predicted by the simulated distribution of isothermal lines (see Fig. 2a). These exotic behaviors thus demonstrate that the thermal metamaterial concentrator is capable of efficiently focusing heat fluxes to desired areas by compressing thermal energy propagation without disturbing the temperature profile outside the device. The same properties can also be observed when the two point thermal sources are placed nearby the two boundaries of the device as shown in Fig. 3c,d. Both strong compression of the isothermal lines in the annulus and generation of constant temperature gradients at the inner region can be observed in the simulated and measured temperature profiles, further evidencing the versatility of the metamaterial device.

To quantify the experimental concentration efficiency and temperature perturbation, we recorded the temperatures at the positions A-G from Fig. 3b, with results shown in Table 1 The temperature at the center of the device is simply calculated as the average temperature of the positions E and F. On the one hand, using the aforementioned equation, we have obtained an experimental efficiency of 83.1%, slightly smaller the simulated value of 96.3% for the same device parameters, which could be attributed to the imperfections of the experimental device. The applied temperature gradient can be calculated from the temperature difference between the hold and cold sources (100 °C) and the side of the device (140 mm), resulting in a value of 0.714 K/mm. Surprisingly, the temperature gradient at the inner region is calculated to be 1.5 K/mm by using the temperature difference at the positions E and F (~30 °C) and their distance (20 mm), more than two times the initially applied gradient. This remarkable increase in the temperature gradient at the inner region accounts for the observed large concentration efficiency of thermal energy. On the other hand, we have observed that the temperature differences between the positions A and G, B and the center, and C and D are 0.2, 0.35 and 0.4 °C, respectively, further confirming that the metamaterial device is free of distortion to the external thermal profile. These results are beyond the recent theoretical and experimental demonstrations of the bending of heat flux with multilayered oriented composites^32^,^33^. Finally, it is worthwhile pointing out that the measured temperatures at the positions F and G (or D and E) are very close to each other, indicating a very small amount of thermal energy losses in the metamaterial compression layer.

## Conclusion

In this work, we have theoretically designed and experimentally fabricated a metamaterial based thermal energy concentrator with possible harvesting efficiency up to unity and without incurring perceivable distortion to the external thermal profile. Our design method is based on the sophisticated transformation thermodynamics with assistance by the numerical finite-element method. The experimental concentrator device is made of a single hollow cylinder-shaped composite metamaterial that has highly anisotropic effective thermal conductivities in the radial and azimuthal directions. The heat fluxes can be effectively guided by the metamaterial compression layer to the central concentration region that exhibits a significantly increased temperature gradient compared to the applied value. More excitingly, the temperature profile outside the device is perfectly preserved without incurring any distortion. We believe that these unprecedented results may open up new possibilities for engineering thermal materials with desired properties that may find plenty of opportunities for dramatically enhancing the efficiency of the existing solar thermal collectors.

## Additional Information

**How to cite this article**: Chen, F. and Yuan Lei, D. Experimental Realization of Extreme Heat Flux Concentration with Easy-to-Make Thermal Metamaterials. *Sci. Rep.* **5**, 11552; doi: 10.1038/srep11552 (2015).

## Figures and Tables

**Table 1:** Temperatures captured from Fig. 3b at positions shown in Fig. 2b.

Position	A	B	C	D	E	F	G	Centre
Temperature °C	53.1	34.8	16.9	17.3	20.2	50.1	53.3	35.2
